# The Neural Mechanisms of Social Learning from Fleeting Experience with Pain

**DOI:** 10.3389/fnbeh.2016.00011

**Published:** 2016-02-12

**Authors:** Yang-Teng Fan, Chenyi Chen, Yawei Cheng

**Affiliations:** ^1^Institute of Neuroscience, National Yang-Ming UniversityTaipei, Taiwan; ^2^Department of Sociology, National Chengchi UniversityTaipei, Taiwan; ^3^Department of Rehabilitation, National Yang-Ming University HospitalYilan, Taiwan

**Keywords:** social learning, pain, anterior insular cortex (AIC), dynamic causal modeling, empathy

## Abstract

Social learning is critical for humans to adapt and cope with rapidly changing surroundings. Although, neuroscience has focused on associative learning and pain empathy, the neural mechanisms of social learning through fleeting pain remains to be determined. This functional MRI study included three participant groups, to investigate how the neuro-hemodynamic response and subjective evaluation in response to the observation of hand actions were modulated by first-hand experience (FH), as well as indirect experience through social-observational (SO), and verbal-informed (VI) learning from fleeting pain. The results indicated, that these three learning groups share the common neuro-hemodynamic activations in the brain regions implicated in emotional awareness, memory, mentalizing, perspective taking, and emotional regulation. The anterior insular cortex (AIC) was commonly activated during these learning procedures. The amygdala was only activated by the FH. Dynamic causal modeling further indicated, that the SO and VI learning exhibited weaker connectivity strength from the AIC to superior frontal gyrus than did the FH. These findings demonstrate, that social learning elicits distinct neural responses from associative learning. The ontogeny of human empathy could be better understood with social learning from fleeting experience with pain.

## Introduction

Social learning has been recognized as a powerful and evolutionarily derived mechanism for assisting humans and animals in adapting and coping with their rapidly changing surroundings (Plotkin and Odling-Smee, 1981; Öhman and Dimberg, 1984; Bandura, 1986; Öhman and Mineka, 2001; Sweller and Sweller, 2006). Although neuroscience has focused on learning and empathy, little is known about the neural mechanisms of social learning whereby an individual can learn feelings of concern for others who suffer in the real world.

The neural mechanisms of social learning have been examined by classical fear conditioning (Phelps et al., 2001; Olsson and Phelps, 2004, 2007; Galef and Laland, 2005; Phelps and LeDoux, 2005). This amygdala-centered model (Olsson et al., 2007) posited, that indirectly attained fears could be as powerful as fears originating from direct experiences. The conditioned stimulus-unconditioned stimulus (CS-US) contingency is expressed in the amygdala, hippocampus, anterior cingulate cortex (ACC), and anterior insular cortex (AIC) when direct experiences and social learning take place. While fears can be acquired through observation, the strength of the CS-US association may be modified by input from the medial prefrontal cortex (mPFC), which is associated with thinking about one's own and others' mental status. As for another social transmission procedure, verbal communication learning is relied on the left-lateralized cortical network. Fear conditioning is quite predictable in which a series of trials are separated by short inter-trial intervals. In the real world, however, the same stimulus is unlikely to repeatedly link to the same outcome within such a short period (Parsons and Davis, 2012).

The overlap between the perception of others' pain and first-hand experience (FH) of pain has been interpreted as a neural mechanism of empathy by which one may share the pain of another (Decety, 2011a). A large body of neuroimaging research revealed that perceiving other individuals in physical pain elicit neurohemodynamic responses in a restricted number of brain regions, including the AIC, ACC, somatosensory cortex (SI/II), and brainstem (see Lamm et al., 2011 for a meta-analysis). These brain regions were also consistently activated by acute physical pain, belonging to the so-called “pain-matrix.”

The pain-matrix may mediate aversive learning (Papini, 2002; Tucker et al., 2005). Lesion of the ACC substantially impairs observational fear learning in mice (Jeon et al., 2010). The AIC is involved in learning the associations between stimuli and outcomes (Ploghaus et al., 1999). Anticipation of pain activates the AIC, which provides information regarding aversive body states in relation to conditional stimuli (Paulus and Stein 2006). Hence, the pain system is not only essential to the modulation of emotionally negative events, but also linked to the cognitive and affective processes that occur during learning (Nitschke et al., 2006; Paulus and Stein, 2006).

**Figure 1:**
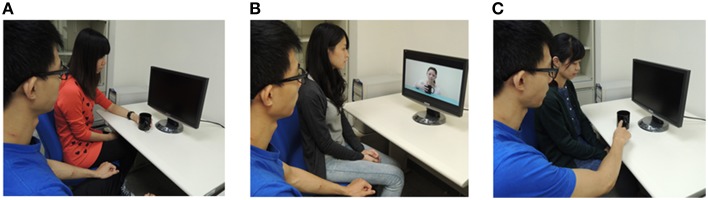
**Direct experiences and social learning involving pain**. Participants learn the association of the stimuli and pain through **(A)** Touching the heated mug (FH). **(B)** Viewing a learning model's expression of distress (SO). **(C)** Verbal information about its aversive qualities (VI).

In response to action observation, we scanned fMRI before and after direct experiences and indirect social transmissions of fleeting pain (Figure 1). During the interval between the first and second scanning, participants left the scanner and were randomly assigned to one of three learning groups. There were two kinds of mugs: one was heated (75°C) and the other was non-heated (30°C). In the FH group, participants touched the mug to learn whether the mug was heated or not. In the social-observational (SO) group, participants watched an animation, in which an actor showed a painful expression when holding a heated mug (H) and a neutral expression when holding a non-heated mug (N). In the verbal-informed (VI) group, the experimenter told participants, that the white-colored mug was heated and the black-colored mug was non-heated. We hypothesized that, if learning through fleeting pain were centered in the amygdala, then various learning procedures would have the common activation in the amygdala. Alternatively, learning indirectly through SO, VI, and FH would recruit the pain matrix, such as ACC and AIC. Furthermore, we explored the effectively causal connections among various learning strategies.

## Materials and methods

### Participants

Fifty-four healthy female volunteers, aged between 20 and 30 years, participated in the study after providing written informed consent. They were mainly recruited from campuses and paid for their participation. All of the participants exhibited normal or corrected-to-normal vision, and were free of neurological and psychiatric symptoms or signs. Considering sex differences in emotional memories and pain empathy (Manstead, 1992; Yang et al., 2009), we enrolled female participants only. The participants were divided into three learning groups, whose age, handedness, IQ, and pressure-pain thresholds (PPT) were matched to each other (Table 1). The study was approved by the local ethics committee (Yang-Ming University Hospital) and conducted in accordance with the Declaration of Helsinki.

**Table 1:** **Demographic data of the study participants**.

	**FH group *n* = 18**	**SO group *n* = 18**	**VI group *n* = 18**	***p***
Age	24.8 ± 3.3	25.4 ± 3.1	25.2 ± 3.2	0.78
FIQ	106.2 ± 4.5	105.8 ± 4.1	106.6 ± 3.7	0.77
PPT (kg/cm^2^)	2.62 ± 0.14	2.56 ± 0.17	2.49 ± 0.27	0.17
Handedness	R∕L(17∕1)	R∕L(17∕1)	R∕L(18∕0)	0.60

FH, first-hand experiences; SO, social-observational; VI, verbal-informed; FIQ, full-scale intelligence quotient; PPT, pressure-pain threshold.

### Procedures

Before fMRI scanning, each participant underwent assessments of handedness, IQ, and PPT. Given the fact that the pain threshold might have an impact on the perception of other's pain (Cheng et al., 2012; Fan et al., 2014), we assessed the PPT, indexing the sensitivity of peripheral nociceptors. The fMRI scanning comprised two sessions with a functional activation paradigm, in which participants were asked to watch the stimuli. During the interval (~10 min) between the first and second fMRI scanning sessions, participants left out the MRI scanner to be randomly assigned to one of three learning groups: FH, SO, or VI. Immediately after the learning procedures, participants were presented with the stimuli they had viewed during the first fMRI scanning and were asked to evaluate the subjective unpleasantness by using a computerized version of the Facial Pain Scale-Revised (FPS-R) (Bieri et al., 1990; Li et al., 2007). The experimenter was always a male.

### Visual stimuli

The stimuli had 12 animations, depicting an actor's right hand approaching and holding the white or black mug. Each stimulus consisted of the successive presentation of three digital color pictures, which were edited to the same size (205 × 154 pixels). The duration of the first, second, and third pictures was 1000, 100, and 1000 ms, respectively. The mugs had the same size and shape. Six actors (three of whom were females) were involved in the presentations, and their facial expressions were not shown.

### Pressure pain threshold (PPT)

The PPT was assessed using a hand-held pressure algometer (UTECH pain track, Zevex international, Salt Lake City, UT, USA), which consists of an ergonomic dynamometer with a flat stainless steel circular probe (1.52 cm^2^) connected to a commander console. The algometer was placed perpendicularly against the skin. The PPT value was determined by applying gradual pressure at the dorsal side of the right and left proximal phalanx of the index finger until the subject reported feeling pain. This procedure was repeated three times, alternating left and right sides, in order to improve reliability. The mean of the six evaluations was calculated for the PPT value.

### MRI data acquisition, imaging processing, and statistical analysis

Structural and functional MRI data were collected using a 3T MRI scanner (Magnetom Tim Trio, Siemens, Erlangen, Germany) equipped with a high-resolution 12-channel head array coil. Changes in blood oxygenation level-dependent (BOLD) T2^*^ weighted MR signal were measured using a gradient echo-planar imaging (EPI) sequence (repetition time TR = 2600 ms, echo time TE = 30 ms, FOV = 220 mm, flip angle = 90°, 64 × 64 matrix, 36 slices/slab covering the whole brain, slice thickness 3 mm, no gap). For each run, a total of 98 EPI volume images were acquired along the AC-PC plane. Structural MR images were acquired with a MPRAGE sequence (TR = 2530, TE = 3.5, FoV = 256 mm, flip angle = 7°, 256 × 256 matrix, 176 slices/slab, slice thickness = 1 mm, no gap).

A factorial design with one between-group factor, learning group (FH vs. SO vs. VI), and two within-group factors, (1) session (first vs. second fMRI scanning), and (2) stimulus (heated mug, H vs. non-heated mug, N), were tested. Each scanning session consisted of two functional runs. Each run included two condition blocks and three fixation blocks. Each condition block (duration 15.6 s each) consisted of six trials that belonged to the same stimulus category (2.1 s each), and six inter-stimulus intervals (500 ms each) with a white fixation presented against a gray background. A 20.8-s fixation block was inserted at the beginning, after each condition block, and at the end of each run. The order of the stimulus conditions was randomized within each run. The sequence and order of the runs and the blocks were counterbalanced across the participants.

Image processing was conducted using SPM8 (Wellcome Department of Imaging Neuroscience, London, UK). The first four volumes of each functional session were discarded to allow for T1 equilibration effects. The remaining images underwent preprocessing, including reorientation, slice-timing correction, correction for head motion, normalization to the EPI template with a resampled voxel size of 3 × 3 × 3 mm, and smoothing with an isotropic 10-mm full-width half-maximum (FWHM) Gaussian Kernel. A two-stage general linear model was used to examine the effect sizes of each condition and to compare them at the group level. At the first level, we created the images of parameter estimate for the following contrasts in each subject:

1. The session effect to identify the brain regions involved in learning procedures, irrespective of the mugs: the second session (H + N) − the first session (H + N).
2. The stimulus effect to identify the brain regions involved in processing pain, irrespective of the sessions: the (first + second) session H − the (first + second) session N.
3. The session-by-stimulus interaction, which was assumed to be associated with the processing of learning procedures regarding pain effect: the second session (H − N) − the first session (H − N).

A statistical threshold of *p* = 0.05, false discovery rate (FDR) corrected, was used for the whole brain analysis. Activations were overlaid on a representative high-resolution structural T1-weighted image from a canonical image set, coregistered to the Montreal Neurological Institute space.

### Region of interest (ROI) definition and analysis

Using the MarsBar toolbox, we defined the ROIs as a 6-mm spherical region centered on the following MMI coordinates, including the SI/II (*x* − 41, *y* − 43, *z* 60), amygdala (−20, −2, 24), AIC (−40, 22, 0), ACC (−2, 23, 40), and superior frontal gyrus (SFG: −24, −8, 62). These coordinates were determined on the basis of neuroanatomical atlases as well as one meta-analyses regarding pain empathy (Lamm et al., 2011). The average beta estimates of all the voxels in these ROIs were analyzed using an analysis of variance (ANOVA) to test session-by-stimulus interactions [the second session (H – N) – the first session (H – N)] at the group level. A Bonferroni correction was applied to account for multiple comparisons.

### Dynamic causal modeling (DCM)

Effective connectivity analyses were conducted using the dynamic causal modeling (DCM) toolbox. Functional imaging data were remodeled at the first level for each subject and for each session. The general linear model for DCM consisted of two regressors, encoding the visual and the heated-mug trials during the second fMRI scanning. DCM was constructed for each subject, involving the four left-lateralized ROIs: the superior temporal sulcus (STS), AIC, ACC, and SFG. After determining the optimal model, we extracted the parameter representing the modulatory effect of aversive learning from the winning model. We used a statistical threshold of *p* < 0.05 (corrected) for effective connectivity analyses.

#### Extracting the time series

Treating the brain as an input-state-output system, DCM estimates how (output) hemodynamic activity from a given brain region depends on (input) variables manipulated in an experiment. These ROIs were defined as 8 mm-radius spheres and were extracted at peak effects for each subject-specific statistical parametric map at *p* = 0.05. Two *t*-contrasts (visual and the heated-mug/2nd trial) were defined in order to extract the time series. One “effect of interest” *F*-contrast was defined for the global mean correcting of the extracted time series. Primary eigenvariate values were drawn from individually selected ROIs from each subject.

#### Model specification and bayesian model selection

The four ROIs were then fed into separate DCMs for each session with each subject. Six models were constructed in order to determine the fittest in terms of connectivity. The intrinsic connectivity was based on anatomical structure, namely reciprocal connection between SFG-AIC, AIC-ACC, AIC-SFG, and ACC-SFG (Cauda et al., 2011). In Model 1, we proposed that the heated-mug situation would modulate the connectivity from AIC to ACC. In Model 2, the heated-mug situation would modulate the connectivity from AIC to SFG. In Model 3, the heated-mug situation would modulate all reciprocal connectivities across the model areas. In Model 4, the heated-mug situation would modulate the connectivities between AIC-SFG, AIC-ACC, SFG-AIC, and ACC-AIC. In Model 5, the heated-mug situation would modulate the connectivity from SFG to AIC. In Model 6, the heated-mug situation would modulate the connectivity from ACC to SFG.

## Results

### Behavioral results

The ANOVA of subjective unpleasantness ratings on the stimuli immediately after learning procedures showed main effects for group [*F*_(2, 51)_ = 17.76, *p* < 0.001] and stimulus [*F*_(1, 51)_ = 74.24, *p* < 0.001]. Participants felt increased unpleasantness toward the heated mugs relative to the non-heated mugs, suggesting that the manipulation of fleeting experiences with pain can be effective. In addition, there was an interaction of group and stimulus [*F*_(2, 51)_ = 17.76, *p* < 0.001]. *Post-hoc* tests indicated that the FH group had more unpleasantness ratings to the heated mugs than the SO (*p* = 0.016) and VI (*p* < 0.001), but none was in the non-heated mugs.

### fMRI results

Irrespective of the stimulus, the session effect among groups [the second session (H + N) – the first session (H + N)] indicated significant activations in the regions implicated in emotional awareness, perspective taking, and emotional regulation. These included AIC, TPJ, ACC, and SFG. However, the inferior frontal gyrus (IFG), a region belonging to the mirror neuron system, was activated in the FH group, but not in the SO and VI. Direct comparisons between learning groups revealed that the FH relative to the SO exhibited a signal increase in the SFG, ACC, and posterior cingulate cortex (PCC). When compared with the VI, the FH group showed stronger signals in the ACC, SFG, mPFC, IFG, and AIC, whereas the SO was associated with signal changes in the SFG, ACC, and mPFC (Table S1).

Irrespective of the session, the stimulus effect among groups [the (first + second) session H – the (first + second) session N] showed activations in the ACC, precentral gyrus, and SI/II. Direct comparisons among groups revealed that the FH group, relative to the SO, showed increased activations in the left precentral gyrus, right IFG, and left TPJ, whereas weaker activations were observed in the midbrain and left superior temporal gyrus. When compared with the VI group, the FH group showed stronger activations in the SI/II, TPJ, and AIC, whereas the SO was associated with a signal increase in the SI/II, precentral gyrus, ACC, and SFG. In particular, the VI group, relative to the FH group, showed stronger activations in the SFG, PCC, precentral gyrus, and thalamus. The VI relative to the SO did not reveal any significant activation (Table S2).

**Figure 2:**
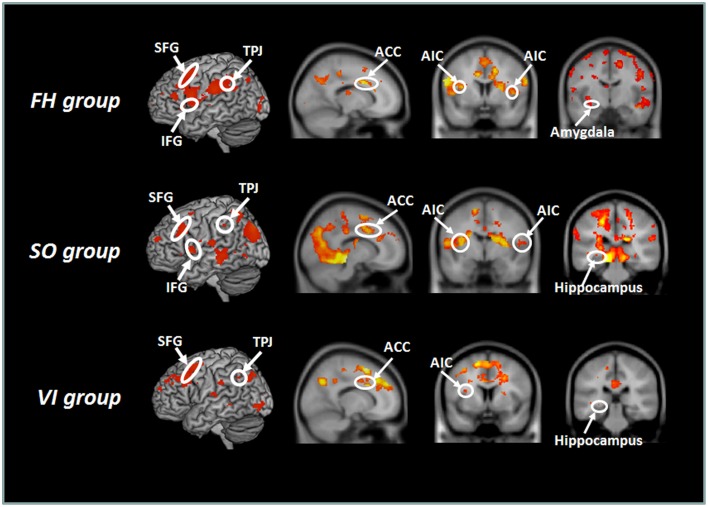
**Hemodynamic responses to the session-by-stimulus interaction among the three learning group: [the second session (H – N) – the first session (H – N)]**. Participants from the SO, FH, and VI groups exhibited significant activations in the anterior insular cortex (AIC), ACC (anterior cingulate cortex), temporoparietal junction (TPJ), superior frontal gyrus (SFG), and hippocampus. The amygdala was activated only in the FH group.

When the session-by-stimulus interaction [the second session (H – N) – the first session (H – N)] were explored, participants from the SO group, as well as the FH and VI groups, exhibited significant activations in the regions implicated in emotional awareness, mentalizing/perspective taking, emotional regulation, and memory/learning (Figure 2). These included the AIC, ACC, TPJ, SFG, and hippocampus. However, the amygdala activation was detected only in the FH group. Furthermore, the SI/II was activated in the FH and SO groups, but not in the VI. Direct comparisons among groups revealed that the FH group, relative to the SO, showed increased activation in the left SI/II and SFG, whereas the reverse comparison revealed significant activations in the left precuneus and right TPJ. When compared with the VI group, the FH group showed stronger activations in the bilateral TPJ, left SI/II, right IFG, right AIC, and right hippocampus, whereas the SO group were associated with stronger signals in the right-lateralized areas, including the SI/II, TPJ, AIC, SFG, and hippocampus. In contrast, the VI group, relative to the FH or SO group, showed no significant activation.

To assess the commonalities among three learning groups, a conjunction analysis on the session-by-stimulus interaction [the second session (H – N) – the first session (H – N)] reavealed overlapping activations in the right medial prefrontal cortex (mPFC), left SFG, TPJ, ACC, and AIC (Tables S3, S4).

### ROI analysis

ANOVAs on selected ROIs were reported to have significant group-by-session and stimulus interactions in the left SI/II, amygdala, and SFG, but none in the AIC and ACC. *Post-hoc* tests indicated that the FH relative to the SO had stronger activations in the SI/II and SFG. In comparison with the VI, the FH showed more activation in the SI/II, amygdala, and SFG, whereas the SO showed stronger activations in the SI/II (please see Supplementary Materials for the statistical details).

### Effective connectivity

**Figure 3:**
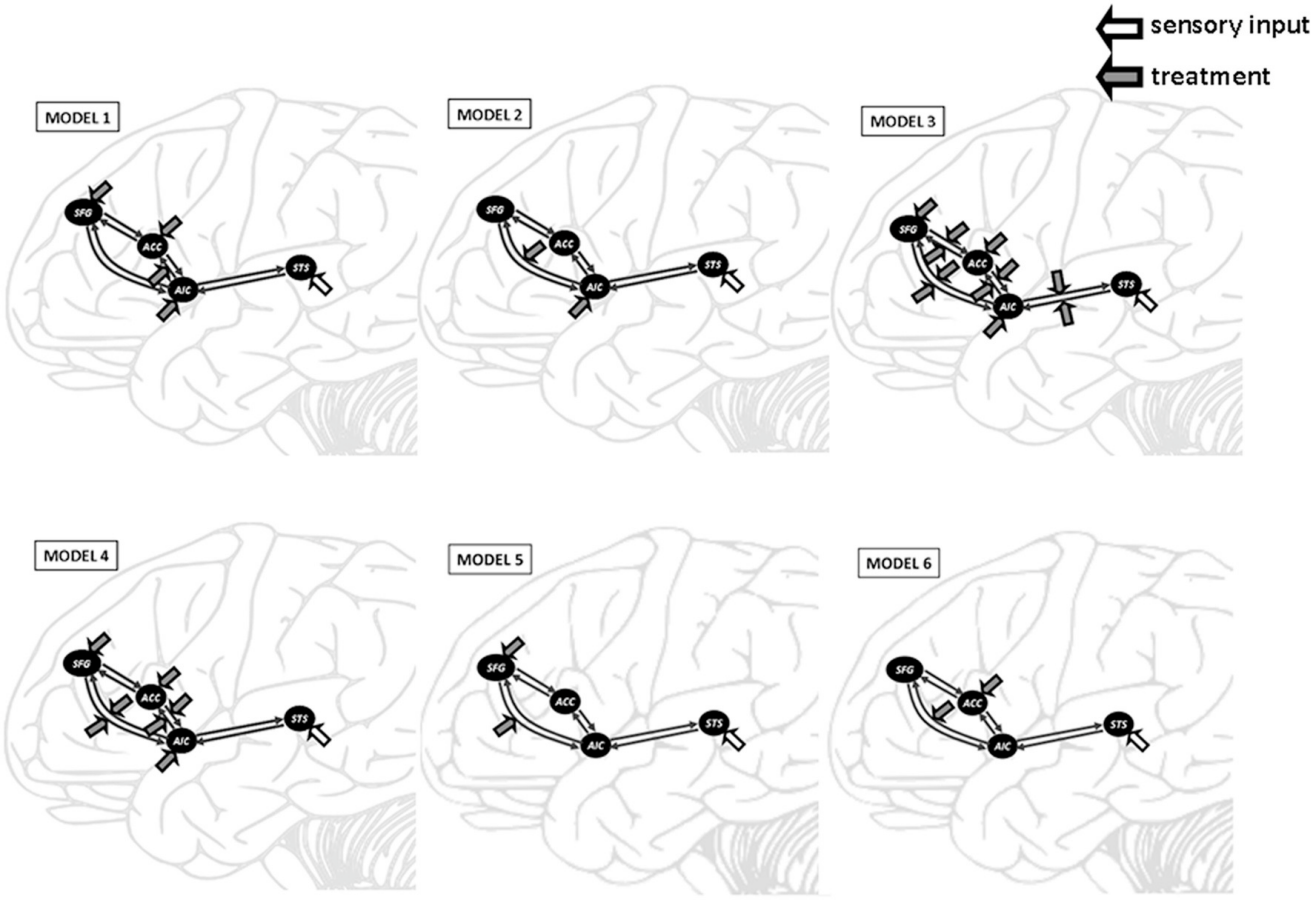
**Outline of the six DCM models tested in this study**. DCM was constructed on the superior temporal sulcus (STS), anterior insular cortex (AIC), anterior cingulate cortex (ACC), and superior frontal gyrus (SFG). The heated-mug situation was proposed to modulate the AIC-ACC connectivity in Model 1, the AIC-SFG connectivity in Model 2, and all reciprocal connectivities in Model 3. Model 4 proposed that the heated-mug situation would modulate the AIC-SFG, AIC-ACC, SFG-AIC, and ACC-AIC connectivities. The heated-mug situation would modulate the SFG-AIC connectivity in Model 5 and the ACC-SFG connectivity in Model 6.

Bayesian model selection (BMS) was carried out with random effect using standard procedures (Penny et al., 2010). Based on BMS (Figure 3), the expected posterior probabilities for models 1-6 were 0.14, 0.66, 0.0088, 0.0057, 0.0055, and 0.18 for the FH; 0.054, 0.68, 0.011, 0.0035, 0.00061, and 0.21 for the SO; and 0.10, 0.58, 0.022, 0.0048, 0.023, and 0.27 for the VI. The model exceedance probabilities were 0.014, 0.78, 0.0058, 0.010, 0.0008, and 0.19 for the FH; 0.01, 0.71, 0.00013, 0.00087, 0.029, and 0.25 for the SO; and 0.017, 0.64, 0.009, 0.0009, 0.023, and 0.31 for the VI.

**Figure 4:**
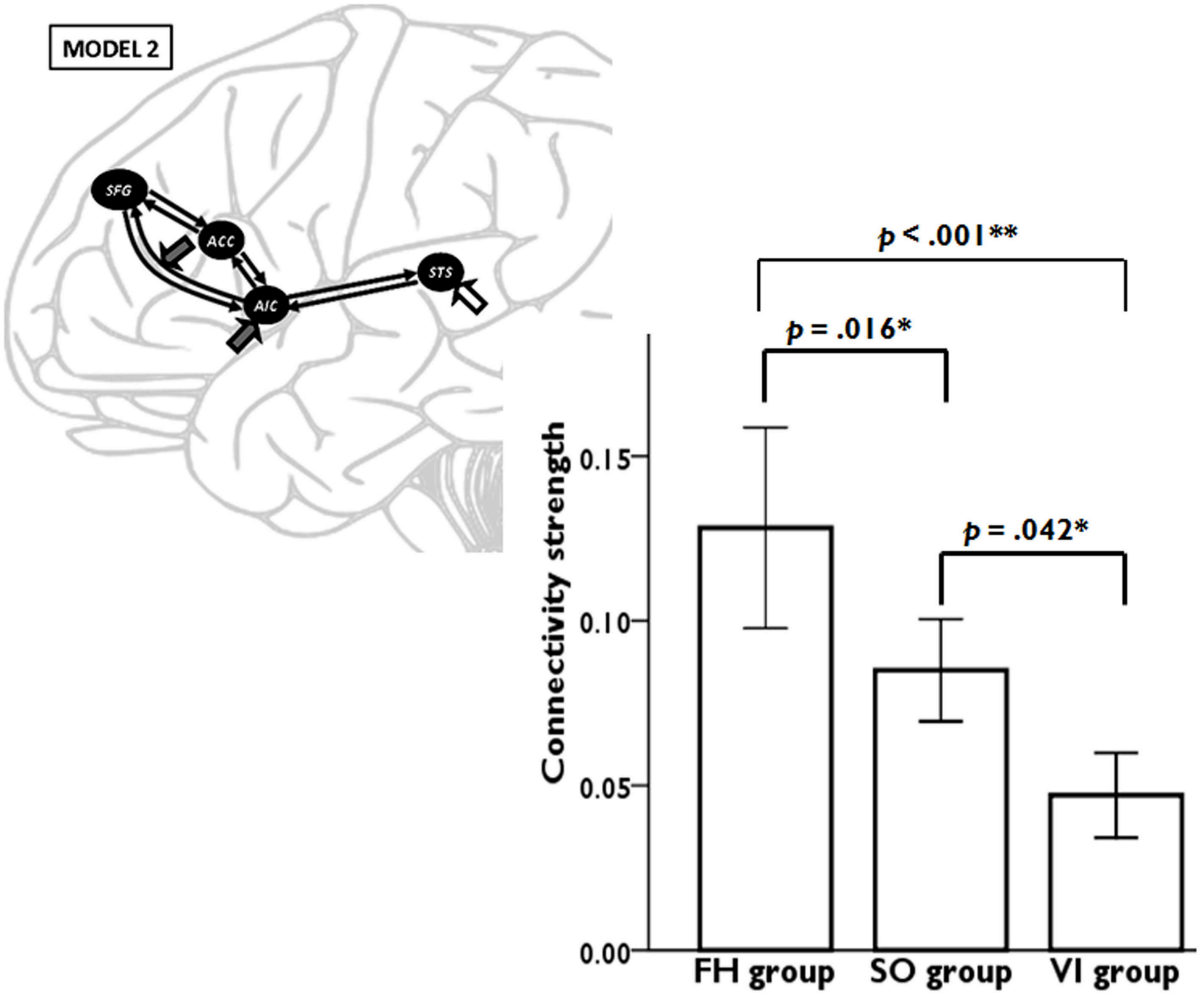
**DCM results demonstrate the connectivity strength from anterior insular cortex (AIC) to superior frontal gyrus (SFG) among three learning groups during the second fMRI session**. Error bars represent standard errors.

Model 2 was superior to the other models among all three learning groups, suggesting, that learning experience exerted its modulatory effect through the connectivity from AIC to SFG. We conducted a one-way ANOVA to compare the AIC-SFG connectivity strength among three learning groups during the second fMRI session. The results indicated that the SO group had less connectivity strength than did the FH (*p* = 0.016). Moreover, the FH and SO groups had more connectivity strength than did the VI (*p* < 0.001; *p* = 0.042; Figure 4).

## Discussion

Although social neuroscience focused attention on the neural underpinnings of pain empathy, little is known about the roles of social learning whereby an individual can acquire and learn feelings of concern for others who suffer in the real world. We investigated various types of aversive learning after fleeting pain and elucidate the neural mechanisms involved in human social learning.

Using a realistic paradigm, we demonstrated that various learning procedures of pain produce similar neural responses. Essentially, both the exploratory and confirmatory analyses indicated that the AIC plays a critical role among three learning procedures. In addition, each procedure recruited other non-overlapping neural regions. These differential activations seem related to distinct functional processes that occur during the FH, SO, and VI acquisition (Olsson and Phelps, 2004). The amygdala was only activated in FH learning. Participants undergoing SO learning exhibited brain activations in the affective component of pain, which was similar to people learning through FH, but demonstrated weaker activation in the sensory discrimination of the pain matrix. Nevertheless, VI learning indicated less activation and fewer connections in neural substrates of pain processing.

The SO learning exhibited compatible neural activations in regions implicated in emotional awareness/understanding, memory, perspective taking, and emotional regulation. The shared neural representations of an individual's emotional experience and perception of the corresponding emotion in another individual are critical to emotional understanding and to empathizing with others (Preston and de Waal, 2002; Gallese et al., 2004). Numerous studies have indicated that the AIC and ACC encode the affective-motivational aspects of pain (Peyron et al., 2000; Critchley et al., 2005) particularly when people observe another person's pain (Singer et al., 2004; Cheng et al., 2010). One fMRI study on observational fear conditioning reported activation in both the ACC and AIC during observation of another person receiving shocks paired with a conditioned stimulus, and also when the person being expected to receive shocks accompanying the same stimulus (Olsson et al., 2007). Ploghaus et al. (1999) used the cue-based paradigm and reported that the hemodynamic responses in the ACC and AIC responded to a colored light signaling pain, but not to a color signaling warmth. Understanding the circumstances, that cause distress in another individual involves taking the other person's perspective, which can trigger empathic responses in the observer (Olsson and Phelps, 2007). In animals, fear can be acquired through social observation of others suffering from aversive stimuli (John et al., 1968; Mineka et al., 1984; Kavaliers et al., 2001). When lidocaine was injected into the ACC, observer mice showed impaired observational fear learning compared with control mice (Jeon et al., 2010). Here, the AIC-SFG connectivity yielded better causal inference than did other connections after three learning procedures. These results supported the notion that the AIC and ACC play a crucial role in social learning.

In spite, of distinct neural activation patterns, VI learning could engage similar neural mechanisms to those evoked by direct experience and by indirect social observation. Similarly to FH and SO, VI learning elicited hemodynamic responses in brain regions associated with the pain system, but these activations showed a left-lateralized tendency. Learning based on language depends on awareness (Phelps et al., 2001; Olsson and Phelps, 2004), likely involving more explicit representations (Olsson and Phelps 2007). The difference in laterality of brain activation might reflect the extent to which participants elaborated and interpreted the representation elicited by aversive stimuli (Phelps et al., 2001). Moreover, the VI relative to FH and SO evoked weaker hemodynamic responses in the SI/II, AIC, and SFG. The neural connectivity from AIC to SFG was weaker than those from the other learning procedures. Learning through FH and SO can immediately elicit a negative representation that are not dependent on higher cortical awareness, whereas aversive learning through a verbal route, during which participants must generate a mental representation of the aversive event, does not exist in the immediate context (Phelps et al., 2001). Accordingly, the results suggested that learning acquired through linguistic inputs, requiring more abstract representations, relies on a neural network distinct from the other learning forms.

The SO and VI groups exhibited less activation in the left SI/II and SFG than did the FH. The FH participants were required to touch the mugs with their own hands. Neurophysiological evidence for nociceptive information processing indicates the involvement of SI/II (Decety, 2007; Liang et al., 2011). During FH, SI/II representation might be primed by directly experiencing actual pain. Alternatively, SI/II representation might be primed by the observation of another individual's emotional display or by abstract instruction during SO and VI learning (Olsson and Phelps, 2007). The SO and VI relative to the FH group did not elicit comparable SI/II activation. The regulation of internal emotional states and processes is particularly relevant to the modulation of vicarious emotion and the experience of empathy (Decety, 2011b). The SFG has been previously linked to the self-regulation of emotions (Cheng et al., 2007; Decety, 2007). Our results demonstrated, that the SFG was involved in the SO and VI, but less activated than in the FH. Based on the modulatory effects on the DCM, the SO, and VI exhibited weaker connectivity from AIC to SFG. Accordingly, we assumed that the SFG activation is modulated by the neural connections through a bottom-up mechanism, which affects regulatory processing during social transmission learning.

The major discrepancy between our results and previous reports (Phelps et al., 2001; Olsson and Phelps, 2004, 2007) was the absence of amygdala activation among various learning procedures. Recent animal studies have reported that a single pairing of a light with a weak shock primes future learning, so that a second trial would result in the formation of a long-lasting memory (Parsons and Davis, 2012). We used the picture-based paradigm based on fleeting exposure to aversive stimuli, rather than repeated CS-US associations where it is likely that the same stimulus predicts the same outcome repeatedly. In the learning model, emotional expressions served as an US and its co-occurrence with the colored squares (CS) was made directly self-relevant to the subject because of its stability to predict future potentially harmful events. The motivation to understand potentially harmful qualities in the surroundings might trigger fear-learning mechanisms, which are known to depend on the amygdala (Phelps and LeDoux, 2005). Furthermore, the neuronal basis of behaviors must consider the connectivity characteristics of functional networks (Passingham et al., 2002). The DCM is considered to be able to assess effective connectivity between brain regions, which can estimate the coupling among brain regions and facilitate the exploration of experimental condition-specific influences of these couplings (Friston et al., 2003). This study combining exploratory and confirmatory analyses can provide more favorable and clear-cut neural evidence in relation to aversive learning.

The limitations of the present study must be acknowledged. Firstly, regarding sample homogeneity, the generalizability of the results may be limited because participants were only females. The experimenter was always a male. The visual stimuli included both male and female actors. Despite, of no statistical significance for the same-gender (female participants/female actors) and opposite-gender (female participants/male actors) interactions in terms of unpleasantness ratings and neuroimaging results, the potential interference of the gender factor in social learning needs to be further clarified. Secondly, only young adults were enrolled. Aging is associated with changes in the neural circuits underlying learning systems and empathy (Dennis and Cabeza, 2011; Chen et al., 2014). Finally, a sample size of only 19 women rendered this study underpowered to detect a statistically significant change. This may not be the optimal design, and future studies in which recruit a larger sample size with female and male subjects across all age groups are warranted.

## Conclusion

Our results revealed the neural mechanisms involved in learning from fleeting pain. Although numerous similarities among the FH, SO, and VI learning emerged, obvious differences were also observed. Social learning elicits distinct neural responses from associative learning. The AIC, a region being a critical hub to integrate salient stimuli and events with visceral and autonomic information (Menon and Uddin, 2010), played a pivotal role in emotional learning. The ontogeny of human empathy could be better understood with learning from fleeting experience with pain. Our findings might benefit future studies exploring the pathological mechanisms associated with socioemotional disturbances and assist with improving knowledge in the area of social transmission of emotional learning.

## Author contributions

YF, CC, and YC took part in designing the study. YF and CC run the experiment and undertook the statistical analysis. YF and YC managed the literature search and wrote the first draft of the manuscript. All authors have contributed to and approved the manuscript.

### Conflict of interest statement

The authors declare that the research was conducted in the absence of any commercial or financial relationships that could be construed as a potential conflict of interest.
