# Supplementary material for: The Neural Mechanisms of Social Learning from Fleeting Experience with Pain
Source: Front Behav Neurosci. 2016 Feb 12;10:11. doi: 10.3389/fnbeh.2016.00011 (PMC4751358; doi:10.3389/fnbeh.2016.00011)
Supplement: Supplementary file 1 [file Table1.DOC]

**SUPPLEMENTARY MATERIALS**

**Behavioral Results**

We tested the power of the study using the G-power 3.0.10 (Faul et al., 2007). The ANOVA of subjective unpleasantness ratings on the stimuli immediately after learning procedures indicated the main effects for group [*F* (2, 51) = 17.76, *p* < 0.001, *η^2^* = 0.41, power = 1.00] and stimulus [*F* (1, 51) = 774.24, *p* < .001, *η^2^* = 0.94, power = 1.00]. Participants felt increased unpleasantness toward the white mugs relative to the black mugs, suggesting that the manipulation of fleeting experiences with pain can be effective. In addition, there was an interaction of group and stimulus [*F* (2, 51) = 17.76, *p* < 0.001, *η^2^* = 0.41, power = 1.00]. Post hoc tests indicated that the FH group had more unpleasantness ratings to the white mugs than the SO (*p* = 0.016, *d* = 0.99) and VI (*p* < 0.001, *d* = 2.02) groups, but none was detected in the black mug trials.

Faul F, Erdfelder E, Lang AG, Buchner A. (2007): G*Power 3: a flexible statistical power analysis program for the social, behavioral, and biomedical sciences. Behav Res Methods 39:175-191.

**ROI Analysis**

ANOVAs on selected ROIs were reported to have significant group-by-session and stimulus interactions in the left SI/SII [F (2, 51) = 23.73, p < 0.001, η2 = 0.48, power = 1.00], amygdala [F (2, 51) = 7.92, p = 0.001, η2 = 0.24, power = 0.99], and SFG [F (2, 51) = 4.59, p = 0.015, η2 = 0.15, power = 0.94], but none in the left AIC [F (2, 51) = 1.69, p = 0.19, η2 = 0.062, power = 0.96], and ACC [F (2, 51) = 0.99, p = 0.38, η2 = 0.037, power = 0.82]. Post hoc tests indicated that the FH relative to SO had stronger activations in the left SI/SII (p = 0.016, d = 0.79) and SFG (p = 0.028, d = 0.87). In comparison with the VI, the FH showed more activation in the left SI/SII (p < 0.001, d = 3.76), amygdala (p = 0.001, d = 1.32), and SFG (p = 0.003, d = 1.81), whereas the SO showed stronger activations in the left SI/SII (p = 0.002, d = 1.17). However, the AIC and ACC were not differentially activated among the three groups (supplementary Figure s1).

**Table S1.** Group differences in brain regions showing the session effect: [the second session (H + N) – the first session (H + N)].

|  | MNI Coordinates | | | *Z* score | Cluster size  (mm^3^) |
| --- | --- | --- | --- | --- | --- |
| Brain area | x | y | z |
| ***FH group*** |  |  |  |  |  |
| R Superior frontal gyrus | 10 | 12 | 56 | 5.12 | 628 |
| R Anterior cingulate cortex | 14 | -2 | 48 | 4.64 | 536 |
| R Tempoparietal junction | 46 | -48 | 42 | 4.03 | 45 |
| L Anterior cingulate cortex | -16 | 4 | 42 | 4.21 | 64 |
| L Precentral gyrus | -42 | 18 | 34 | 5.00 | 349 |
| L Precuenus | -18 | -58 | 32 | 4.01 | 26 |
| L Temporparietal junction | -64 | -24 | 28 | 5.48 | 419 |
| R Precentral gyrus | 60 | 4 | 28 | 5.01 | 120 |
| R Lentiform | 26 | 0 | 18 | 5.45 | 448 |
| L Inferior frontal gyrus | -52 | 10 | 16 | 6.05 | 555 |
| L Middle frontal gyrus | -38 | 34 | 16 | 5.41 | 549 |
| R Middle frontal gyrus | 28 | 40 | 14 | 4.45 | 69 |
| R Anterior insula cortex | 36 | 14 | 8 | 4.59 | 442 |
| L Anterior Insula cortex | -46 | 8 | 4 | 5.29 | 555 |
| L Lingual gyrus | -18 | -92 | -2 | 4.66 | 74 |
| ***SO group*** |  |  |  |  |  |
| L Superior frontal gyrus | -14 | -2 | 62 | 3.88 | 42 |
| R Superior frontal gyrus | 6 | 14 | 62 | 3.27 | 13 |
| L Precuneus | -16 | -46 | 48 | 3.63 | 253 |
| L Anterior cingulate cortex | -12 | 0 | 42 | 4.41 | 508 |
| R Temporparietal junction | 62 | -40 | 42 | 3.62 | 20 |
| L Temporparietal junction | 62 | -40 | 40 | 3.47 | 35 |
| R Anterior cingulate cortex | 8 | 24 | 38 | 3.77 | 505 |
| R Middle temporal gyrus | 28 | 58 | 22 | 4.90 | 284 |
| L Middle temporal gyrus | -34 | -54 | 14 | 6.40 | 518 |
| R Anterior insula cortex | 38 | 8 | 12 | 4.51 | 491 |
| L Anterior insula cortex | -52 | 8 | 4 | 5.23 | 518 |
| R Precentral gyrus | 52 | 10 | 4 | 4.75 | 491 |
| R Cerebellum | -14 | -30 | -8 | 5.89 | 882 |
| ***VI group*** |  |  |  |  |  |
| L Superior frontal gyrus | -18 | 10 | 56 | 5.77 | 951 |
| L Precuenus | -14 | -30 | 52 | 3.84 | 26 |
| L Precentral gyrus | -28 | -16 | 46 | 4.12 | 20 |
| R Anterior cingulate cortex | 20 | -16 | 44 | 4.83 | 68 |
| L Middle frontal gyrus | -44 | 4 | 42 | 4.55 | 44 |
| L Posterior cingulate cortex | -14 | -46 | 40 | 4.35 | 75 |
| L Temporparietal gyrus | -50 | -48 | 36 | 4.43 | 201 |
| L Anterior cingulate cortex | -28 | 2 | 32 | 4.02 | 43 |
| R Posterior cingulate cortex | 20 | -40 | 32 | 3.24 | 26 |
| R Lentiform | 22 | 8 | 12 | 4.08 | 27 |
| L Thalamus | -10 | -14 | 8 | 4.58 | 202 |
| L Anterior insula cortex | -44 | 6 | 4 | 3.69 | 23 |
| L Lingual gyrus | -4 | -80 | -4 | 4.17 | 57 |
| L Cerebellum | -4 | -42 | -16 | 3.66 | 26 |
| ***FH group > SO group*** |  |  |  |  |  |
| L Temporparietal junction | -44 | -34 | 44 | 4.41 | 114 |
| R Superior fontal gyrus | 20 | 38 | 36 | 3.62 | 15 |
| R Anterior cingulate cortex | 14 | 12 | 30 | 3.63 | 14 |
| R Middle frontal gyrus | 54 | 16 | 30 | 4.11 | 63 |
| L Middle frontal gyrus | -44 | 18 | 32 | 3.75 | 29 |
| L Precentral gyrus | -58 | 4 | 26 | 5.09 | 273 |
| L Lingual gyrus | -18 | -92 | -2 | 5.09 | 194 |
| ***FH group > VI group*** |  |  |  |  |  |
| R Temporparietal junction | 40 | -36 | 48 | 5.92 | 1342 |
| R Precentral gyrus | 54 | 0 | 28 | 5.75 | 1342 |
| L Postcentral gyrus | -64 | -12 | 26 | 5.71 | 198 |
| R Anterior cingulate cortex | 10 | -2 | 44 | 3.73 | 11 |
| R Middle frontal gyrus | 32 | 34 | 28 | 3.71 | 24 |
| R Superior frontal gyrus | 18 | 40 | 30 | 3.62 | 63 |
| R Lingual gyrus | 28 | -74 | 8 | 4.55 | 228 |
| L Lingual gyrus | -32 | -76 | 2 | 4.61 | 300 |
| L Middle temporal gyrus | -44 | -58 | -4 | 4.61 | 300 |
| R Middle temporal gyrus | 40 | -60 | -4 | 3.87 | 122 |
| L Inferior temporal gyrus | -36 | -48 | -10 | 4.47 | 41 |
| ***SO group > FH group*** |  |  |  |  |  |
| R Thalamus | 12 | -26 | -4 | 6.24 | 395 |
| L Posterior cingulate cortex | -2 | -56 | 6 | 5.72 | 395 |
| L Middle temporal gyrus | -34 | -54 | 14 | 5.57 | 244 |
| R Posterior cingulate cortex | 8 | -26 | 22 | 6.03 | 113 |
| R Cerebellum | 2 | -70 | -4 | 4.91 | 26 |
| ***SO group > VI group*** |  |  |  |  |  |
| R Caudate | 16 | -26 | 24 | 4.94 | 11 |
| R Cerebellum | 14 | -80 | 26 | 6.18 | 439 |
| R Anterior insula cortex | 36 | 16 | 18 | 5.76 | 56 |
| L Inferior temporal gyrus | -36 | -46 | -10 | 5.32 | 16 |
| L Postcentral gyrus | -26 | -40 | 66 | 4.93 | 14 |
| L Cerebellum | -22 | -66 | -16 | 5.08 | 12 |
| L Posterior cingulate cortex | -16 | -62 | 12 | 4.90 | 32 |
| ***VI group > FH group*** |  |  |  |  |  |
| none | | | | | |
| ***VI group > SO group*** |  |  |  |  |  |
| none | | | | | |

Peak activation is significant when corrected for multiple comparisons with

False Discovery Rate, p < 0.05.

**Table S2.** Group differences in regions showing the stimulus effect [the (first + second) session H –the (first + second) session N]

|  | MNI Coordinates | | | *Z* score | Cluster size  (mm^3^) |
| --- | --- | --- | --- | --- | --- |
| Brain area | x | y | z |
| ***FH group*** |  |  |  |  |  |
| R Medial frontal gyrus | 14 | 6 | 56 | 5.37 | 220 |
| L Middle frontal gyrus | -30 | 12 | 38 | 4.67 | 129 |
| R Superior frontal gyrus | 20 | 36 | 32 | 5.04 | 145 |
| L Temporparietal junction | -34 | -38 | 30 | 4.49 | 16 |
| L Precuneus | -10 | -68 | 30 | 4.22 | 72 |
| L Superior temporal gyrus | -36 | -48 | 22 | 5.52 | 210 |
| R Posterior cingulate cortex | 14 | -48 | 20 | 4.95 | 75 |
| L Posterior cingulate cortex | -12 | -52 | 18 | 4.02 | 220 |
| R Cerebellum | 4 | -66 | -14 | 5.54 | 419 |
| ***SO group*** |  |  |  |  |  |
| L Precentral gyrus | -24 | -22 | 64 | 5.19 | 144 |
| R Superior frontal gyrus | 2 | 10 | 60 | 4.16 | 18 |
| R Anterior cingulate cortex | 22 | 12 | 28 | 4.76 | 211 |
| L Caudate | -22 | -4 | 26 | 5.79 | 211 |
| L Precuneus | -22 | -38 | 26 | 4.99 | 20 |
| R Middle frontal gyrus | 26 | 28 | 24 | 4.89 | 365 |
| R Caudate | 20 | -12 | 22 | 4.97 | 101 |
| L Anterior insula cortex | -34 | 8 | 18 | 4.83 | 81 |
| ***VI group*** |  |  |  |  |  |
| L Anterior cingulate cortex | -6 | 22 | 32 | 4.37 | 136 |
| R Medial frontal gyrus | 20 | 24 | 30 | 5.34 | 282 |
| L Middle frontal gyrus | -26 | 38 | 26 | 4.25 | 26 |
| L Posterior cingulate cortex | -20 | -52 | 22 | 4.77 | 22 |
| L Anterior insula cortex | -34 | 8 | 18 | 4.83 | 14 |
| L Medial frontal gyrus | -6 | 50 | 16 | 4.26 | 58 |
| ***FH group > SO group*** |  |  |  |  |  |
| L Middle frontal gyrus | -28 | 20 | 46 | 4.14 | 45 |
| R Superior frontal gyrus | 20 | 38 | 36 | 4.21 | 67 |
| L Anterior cingulate cortex | 12 | -8 | 32 | 4.15 | 41 |
| R Posterior cingulate cortex | 14 | -48 | 20 | 5.41 | 193 |
| L Superior temporal gyrus | -36 | -48 | 20 | 4.20 | 31 |
| L Cerebellum | -2 | -58 | 16 | 4.98 | 92 |
| ***FH group > VI group*** |  |  |  |  |  |
| R Postcentral gyrus | 12 | -32 | 58 | 3.85 | 23 |
| L Superior frontal gyrus | -18 | 38 | 38 | 3.82 | 11 |
| L Posterior cingulate cortex | -36 | -38 | 34 | 3.82 | 25 |
| R Precentral gyrus | 56 | -8 | 32 | 3.89 | 43 |
| L Caudate | 14 | -32 | 24 | 5.74 | 220 |
| L Superior temporal gyrus | -36 | -48 | 22 | 4.87 | 103 |
| R Precentral gyrus | 38 | 0 | 22 | 3.72 | 33 |
| R Posterior cingulate cortex | 14 | -48 | 20 | 3.88 | 11 |
| R Anterior insula cortex | 42 | 6 | 16 | 4.24 | 33 |
| R Lingual gyrus | 30 | -62 | 6 | 4.25 | 48 |
| L Cerebellum | -4 | -64 | -12 | 4.73 | 193 |
| ***SO group > FH group*** |  |  |  |  |  |
| L Precuneus | -22 | -54 | 40 | 4.63 | 11 |
| L Posterior cingulate cortex | -22 | -36 | 28 | 6.00 | 11 |
| R Caudate | 20 | -12 | 22 | 4.72 | 16 |
| R Middle frontal gyrus | 30 | 36 | 20 | 4.50 | 12 |
| ***SO group > VI group*** |  |  |  |  |  |
| R Superior frontal gyrus | 16 | -10 | 62 | 3.83 | 15 |
| L Posterior cingulate cortex | -20 | -36 | 28 | 6.41 | 142 |
| R Posterior cingulate cortex | 24 | -40 | 28 | 5.12 | 25 |
| L Superior temporal gyrus | -42 | -42 | 8 | 4.73 | 16 |
| R Cerebellum | 4 | -40 | -10 | 5.01 | 19 |
| ***VI group > FH group*** |  |  |  |  |  |
| R Anterior cingulate cortex | 20 | 22 | 28 | 5.30 | 21 |
| R Middle frontal gyrus | 30 | 36 | 20 | 5.36 | 16 |
| L Thalamus | -20 | -18 | 2 | 5.52 | 14 |
| ***VI group > SO group*** |  |  |  |  |  |
| none | | | | | |

Peak activation is significant when corrected for multiple comparisons with

False Discovery Rate, p < 0.05.

**Table S3.** Group differences in regions significantly activated for the interaction of sessions by stimulus: [the second session (H – N) – the first session (H – N)].

|  | MNI Coordinates | | | *Z* score | Cluster size  (mm^3^) |
| --- | --- | --- | --- | --- | --- |
| Brain area | x | y | z |
| ***FH group*** |  |  |  |  |  |
| L Superior frontal gyrus | -2 | 16 | 56 | 4.61 | 287 |
| R Superior frontal gyrus | 6 | 10 | 56 | 3.94 | 281 |
| L Anterior cingulate cortex | -14 | 8 | 32 | 4.45 | 40 |
| R Anterior cingulate cortex | 14 | 12 | 30 | 5.02 | 153 |
| L Postcentral gyrus | -62 | -22 | 28 | 6.45 | 549 |
| L Temporparietal junction | -62 | -52 | 28 | 3.99 | 549 |
| R Inferior frontal gyrus | 58 | 16 | 22 | 4.88 | 91 |
| L Inferior frontal gyrus | -52 | 10 | 18 | 5.72 | 452 |
| R Anterior insula cortex | 32 | -4 | 18 | 4.98 | 87 |
| R Lentiform gyrus | 26 | 0 | 18 | 5.69 | 263 |
| L Middle frontal gyrus | -40 | 34 | 14 | 4.40 | 44 |
| L Anterior insula cortex | -38 | 6 | 12 | 4.02 | 58 |
| R Hippocampus | 28 | -30 | -4 | 3.19 | 11 |
| L Amygdala | -30 | -10 | -10 | 3.34 | 13 |
| ***SO group*** |  |  |  |  |  |
| L Superior frontal gyrus | -14 | -2 | 62 | 3.96 | 17 |
| R Superior frontal gyrus | 6 | 14 | 58 | 3.58 | 15 |
| L Postcentral gyrus | -18 | -30 | 52 | 4.27 | 106 |
| R Postcentral gyrus | 28 | -22 | 42 | 3.77 | 19 |
| L Posterior cingulate cortex | -4 | -24 | 42 | 3.70 | 54 |
| R Temporparietal junction | 62 | -40 | 42 | 3.64 | 16 |
| R Anterior cingulate cortex | 16 | 10 | 40 | 3.79 | 36 |
| L Anterior cingulate cortex | -4 | 20 | 34 | 4.30 | 298 |
| L Caudate | -22 | -4 | 24 | 5.35 | 753 |
| R Middle frontal gyrus | 28 | 60 | 20 | 5.62 | 383 |
| L Anterior insula cortex | -34 | 6 | 18 | 6.20 | 753 |
| L Middle temporal gyrus | -34 | -54 | 14 | 6.63 | 286 |
| R Anterior insula cortex | 38 | 10 | 14 | 4.58 | 39 |
| L Precentral gyrus | -54 | -4 | 12 | 4.10 | 27 |
| L Lentiform gyrus | -30 | -22 | 6 | 4.38 | 158 |
| L Inferior frontal gyrus | -50 | 10 | 4 | 5.59 | 753 |
| L Thalamus | -20 | -16 | 2 | 4.93 | 158 |
| L Cerebellum | -14 | -30 | -8 | 5.58 | 924 |
| R Hippocampus | 24 | -46 | -10 | 3.34 | 18 |
| ***VI group*** |  |  |  |  |  |
| L Superior frontal gyrus | -2 | 8 | 58 | 4.20 | 208 |
| L Precentral gyrus | -46 | -4 | 46 | 4.39 | 39 |
| L Middle frontal gyrus | -44 | 4 | 42 | 4.32 | 19 |
| L Anterior cingulate cortex | -20 | -4 | 38 | 3.21 | 12 |
| L Precentral gyrus | -30 | 0 | 36 | 4.46 | 42 |
| L Temporparietal junction | -52 | -48 | 36 | 3.78 | 40 |
| R Anterior cingulate cortex | 20 | 16 | 32 | 6.05 | 84 |
| L Anterior insula cortex | -30 | 8 | 20 | 3.65 | 11 |
| L Thalamus | -4 | -22 | 8 | 3.56 | 24 |
| L Hippocampus | -28 | -28 | -6 | 3.19 | 11 |
| R Cerebellum | 2 | -44 | -12 | 4.06 | 43 |
| ***FH group > SO group*** |  |  |  |  |  |
| L Postcentral gyrus | -58 | -22 | 34 | 3.79 | 47 |
| L Superior frontal gyrus | -56 | 6 | 30 | 3.81 | 31 |
| L Middle frontal gyrus | -30 | 20 | 26 | 3.69 | 22 |
| L Lingual gyrus | -22 | -90 | -6 | 3.88 | 16 |
| ***FH group > VI group*** |  |  |  |  |  |
| R Temporparietal junction | 40 | -34 | 50 | 4.29 | 71 |
| L Temporparietal junction | -44 | -32 | 48 | 3.77 | 16 |
| L Precuneus | -16 | -68 | 40 | 3.68 | 49 |
| R Middle frontal gyrus | 36 | 30 | 34 | 4.47 | 98 |
| L Postcentral gyrus | -52 | -20 | 30 | 5.32 | 846 |
| L Superior frontal gyrus | -60 | 6 | 30 | 4.17 | 28 |
| R Inferior frontal gyrus | 58 | 16 | 22 | 5.31 | 370 |
| R Superior frontal gyrus | 20 | 46 | 22 | 3.66 | 19 |
| L Anterior insula cortex | -32 | 20 | 20 | 3.88 | 12 |
| L Precentral gyrus | -54 | -10 | 14 | 4.01 | 41 |
| R Hippocampus | 20 | -40 | 8 | 3.76 | 10 |
| ***SO group > FH group*** |  |  |  |  |  |
| L Precentral gyrus | -28 | -26 | 66 | 4.01 | 25 |
| R Precuneus | 2 | -62 | 54 | 3.69 | 36 |
| L Middle temporal gyrus | -32 | -54 | 14 | 4.58 | 193 |
| R Superior temporal gyrus | 46 | -24 | 8 | 3.82 | 42 |
| L Thalamus | -28 | -28 | 6 | 4.56 | 68 |
| ***SO group > VI group*** |  |  |  |  |  |
| R Superior frontal gyrus | 16 | -12 | 66 | 3.63 | 16 |
| R Postcentral gyrus | 36 | -34 | 60 | 3.82 | 38 |
| R Precuneus | 18 | -44 | 58 | 3.37 | 23 |
| R Temporparietal junction | 52 | -30 | 38 | 3.46 | 35 |
| L Caudate | -28 | -32 | 20 | 4.07 | 20 |
| R Anterior insula cortex | 38 | 16 | 18 | 4.35 | 38 |
| R Hippocampus | 18 | -40 | 8 | 4.22 | 35 |
| L Posterior cingulate cortex | -8 | -40 | 8 | 4.72 | 18 |
| R Cerebellum | 16 | -48 | -10 | 5.48 | 67 |
| L Cerebellum | -20 | -44 | -16 | 5.64 | 57 |
| ***VI group > FH group*** |  |  |  |  |  |
| none | | | | | |
| ***VI group > SO group*** |  |  |  |  |  |
| none | | | | | |

Peak activation is significant when corrected for multiple comparisons with

False Discovery Rate, p < 0.05.

**Table S4.** Overlap among three groups in regions showing the interaction of session by stimulus: [the second session (H – N) – the first session (H – N)].

|  | MNI Coordinates | | | *Z* score | Cluster size  (mm^3^) |
| --- | --- | --- | --- | --- | --- |
| Brain area | x | y | z |
| L Superior frontal gyrus | -2 | 12 | 58 | 3.42 | 41 |
| R Medial frontal gyrus | 14 | 12 | 42 | 2.68 | 12 |
| L Temporparietal junction | -60 | -36 | 28 | 2.69 | 11 |
| L Anterior cingulate cortex | -14 | 14 | 38 | 2.93 | 11 |
| L Anterior insula cortex | -44 | 4 | 4 | 2.63 | 8 |

A threshold of *P* = 0.001 uncorrected with extent threshold = 6
